# Unusually delayed manifestation of a hallux osteoid osteoma: A case report

**DOI:** 10.1016/j.ijscr.2020.02.032

**Published:** 2020-02-19

**Authors:** Meltem Özdemir, Rasime Pelin Kavak, Begüm Demirler Şimşir, Evrim Duman

**Affiliations:** a University of Health Sciences, Dışkapı Yıldırım Beyazıt Training and Research Hospital, Department of Radiology, Ankara, Turkey; b University of Health Sciences, Dışkapı Yıldırım Beyazıt Training and Research Hospital, Department of Orthopaedic Surgery, Ankara, Turkey

**Keywords:** Osteoid osteoma, Hallux, Radiography, Computed tomography, Magnetic resonance imaging, Case report

## Abstract

**Introduction:**

Osteoid osteomas are benign osteoblastic bone tumors mostly seen in patients in the second or third decade of life, and they most frequently involve the femur and tibia. Hallux osteoid osteoma is an extremely rare occurrence with only 14 reported cases to date.

**Presentation of case:**

A 46-year-old woman with a right hallux pain for the last 18 months was admitted. Her foot radiographs showed a small sclerotic focus on the distal phalanx and degenerative changes in the interphalangeal joint of the hallux. The complaints of the patient were attributed to osteoarthritis involving the interphalangeal joint and non-steroidal anti-inflammatory drugs were administered; however, the patient’s condition did not improve, and hallux osteoid osteoma could not be correctly diagnosed until a CT scan was performed.

**Discussion:**

We present a case of hallux osteoid osteoma in which the diagnosis was delayed greatly due to the unusuality of the lesion location, the advanced age of the patient, and the uncertainty of the clinical and radiographic appearance.

**Conclusion:**

Although rare, osteoid osteoma may occur in the hallux. Even if the patient age, pain pattern, and radiographic findings do not exactly meet the classical definitions for osteoid osteoma, this tumor should always be included in the differential diagnosis list in patients presenting with foot pain.

## Highlights

• Osteoid osteoma typically occurs in children or in adolescents. However, it should be noted that this tumor can be detected in older age groups as well.
• Although extremely rare, osteoid osteoma may occur in the hallux.
• CT is the method of choice in the diagnosis of osteoid osteoma.
• Osteoid osteoma should be included in the differential diagnosis list in all patients presenting with foot pain.

## 1 Introduction

Osteoid osteoma (OO) is a benign osteoblastic bone tumor that constitutes 10% of all benign bone lesions [1]. It is a small spherical tumor of no more than 1.5 cm and is characteristically composed of a lucent nidus surrounded by a reactive sclerotic rim. The central nidus contains dilated vessels, osteoblasts, and osteoid. The central nidus levels of prostaglandins, which are believed to cause tumor-related pain, are reported to be 100- to 1000-fold higher than that of normal tissues. The tumor-related pain is typically nocturnal and is relieved using salicylates [2].

OOs typically occur in children, particularly adolescents, and have a male predilection. Most cases are reported to occur in the second or third decade of life [1]. The most common sites of OOs are the long bones of the lower extremity, with approximately half of all OOs involving the femur or tibia. Phalangeal lesions of the foot account for only 2% of all OOs [3]. We herein present an unusual case of a foot OO in the distal phalanx of the hallux that unexpectedly manifested at an advanced patient age. The presentation of this case was prepared in accordance with the SCARE criteria [4].

## 2 Case presentation

**Fig. 1:**
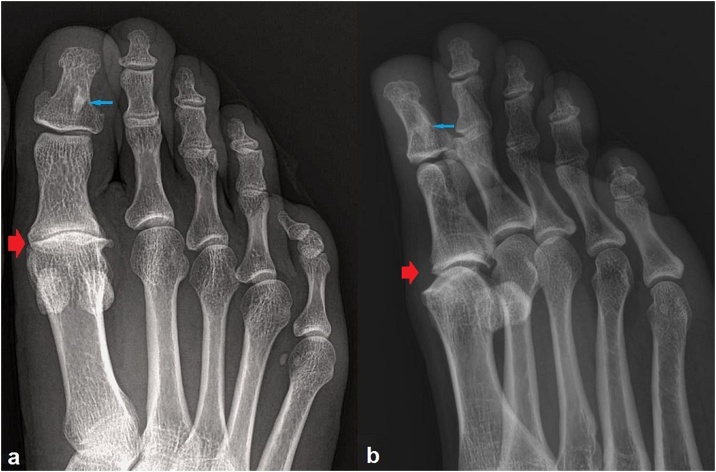
Anteroposterior and lateral-oblique foot radiographs demonstrating an osteoid osteoma in the distal phalanx (blue arrows) and degenerative disease involving the interphalangeal joint (red arrows) of the hallux.

A 46-year-old woman was admitted to the Department of Orthopedic Surgery with the complaint of a right hallux pain for the last 18 months. She had been to a doctor several times, but she had not benefited from the prescribed non-steroidal anti-inflammatory drugs (NSAID). The pain was occurring after long periods of standing and fatigue. There was no history of trauma. Anteroposterior and lateral oblique radiographs of the right foot revealed a cancellous, oval-shaped opacity of 7 × 3 mm in the distal phalanx of the hallux that was interpreted as a bone island or a sclerotic focus of a healed benign bone lesion. A joint space narrowing accompanied by periarticular sclerosis and osteophyte formation in the interphalangeal joint of the hallux was evident in radiographs (Fig. 1). The patient was prescribed an NSAID that she had not tried before, advised to avoid strenuous exercise, and invited for a three months’ control.

**Fig. 2:**
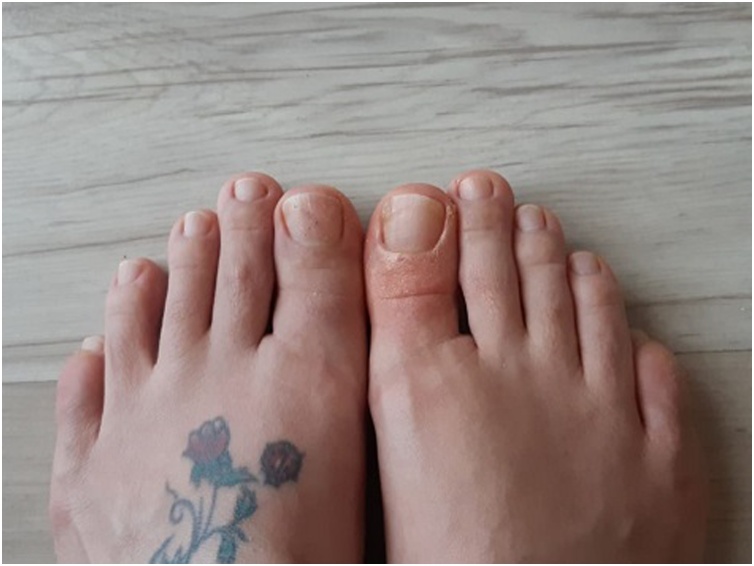
Clinical photograph of the swollen right hallux with erythema.

**Fig. 3:**
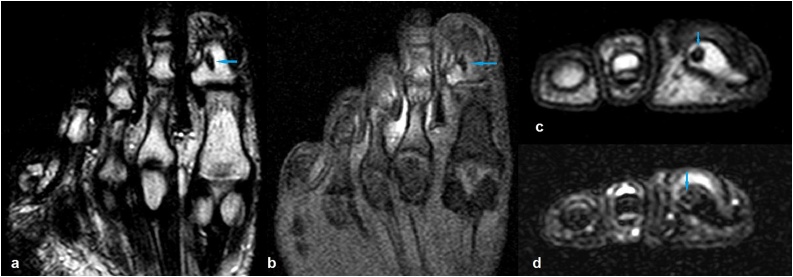
Coronal T1- and fat suppressed T2-weighted (a, b) and axial T1- and fat suppressed T2-weighted (c, d) magnetic resonance images demonstrating an osteoid osteoma in the distal phalanx of the hallux.

**Fig. 4:**
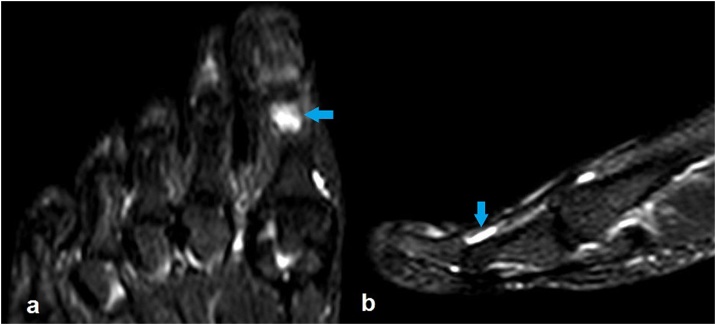
Coronal (a) and sagittal (b) fat suppressed T2-weighted magnetic resonance images showing a slight effusion at the insertion area of the extensor hallucis longus tendon.

**Fig. 5:**
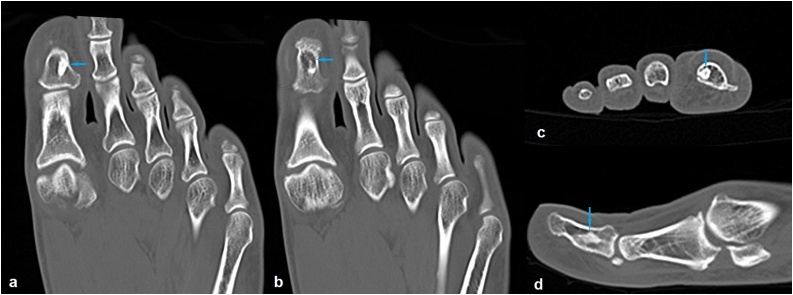
Consecutive coronal (a, b), axial (c) and sagittal (d) computed tomography images demonstrating the typical bony findings of the osteoid osteoma in the distal hallux.

Two months later, the patient was admitted with a progressed and worsened right hallux pain that was unresponsive to the NSAID medication. This time, the finger was swollen with obvious erythema and tender to palpation (Fig. 2). The patient was otherwise well, with an unremarkable complete blood analysis. The radiographic examination findings were identical to those of the prior one (Fig. 1). Magnetic resonance imaging (MRI) of the right foot was performed with the use of a phased array coil. Coronal T1- and fat-suppressed T2-weighted images revealed a signal-free focal cancellous lesion consistent with a sclerotic focus. The lesion was surrounded by an edema that was confined to the distal phalanx and characterized by a high signal on fat-suppressed T2-weighted images (Fig. 3a,b). Axial T1- and fat- suppressed T2-weighted images depicted a relatively high signal in the center of the lesion (Fig. 3c,d). Coronal and sagittal fat-suppressed T2-weighted images demonstrated a slight effusion at the insertion area of the extensor hallucis longus tendon (Fig. 4). Then, a computed tomography (CT) of the right foot was obtained. A radiolucent central nidus surrounded by the characteristic sclerotic rim consistent with OO was obviously visible in the CT images (Fig. 5). Based on the rather specific sectional imaging findings, the lesion was diagnosed as osteoid osteoma.

Following the diagnosis, salicylate treatment was started immediately and a significant decrease was recorded in the patient's complaints. A percutaneous radiofrequency ablation under CT guidance was planned for the treatment of the lesion; however, because the patient feared the planned intervention and was significantly less complaining under salicylate, she requested that the intervention be postponed.

## 3 Discussion

We presented a case of hallux OO in which the diagnosis was delayed greatly due to the unusuality of the case in terms of the lesion location and the patient age as well as the uncertainty of its clinical and radiographic appearances.

Foot and ankle OOs constitute 2–11% of all OOs with a predilection for the talus and calcaneus [5,6]. Hallux OO is an extremely rare occurrence with only 14 reported cases to date [7]. All the previously reported patients with hallux OO presented with a hallux pain and experienced a delay in the diagnosis. The time elapsed between the onset of the symptoms and the diagnosis ranged between 8 and 36 months with a mean of 16 months. Similarly, the diagnosis of our patient was made approximately 20 months after the onset of her pain [7]. The main reason for the delay in the diagnosis of these cases, including that of our patient, was probably that OO was not included in the differential diagnosis list. The most common causes of the hallux pain are trauma, degenerative disease, osteomyelitis, epidermoid inclusion cysts, and tumors (chondrocarcoma, osteoblastoma, subungual squamous cell carcinoma, intracortical osteosarcoma) [8]. During the initial evaluation of the patient, we attributed her pain to the degenerative disease involving the interphalangeal joint; however, to prevent a significant delay in the diagnosis, OO should also be kept in mind as a potential cause of the hallux pain.

OO is classically known as a tumor of childhood and adolescence [1]. According to previous studies, 95% of the patients with OO are under the age of 40 and 80% are under 30 [3]. Similarly, the age of the previously reported patients with hallux OO ranged between 9 and 37, and most of them were in the second or third decade of life [7]. Conversely, it has been reported that, although rare, OO may be detected in patients up to 69 years of age [3]. In the current case, OO manifested in a 46-year-old woman, but an OO diagnosis did not receive early consideration due to the advanced age of the patient. This misinterpretation was one of the factors contributing to the delay in the diagnosis of our patient.

OOs typically present with pain that worsens at night and diminishes under salicylates. Swelling and erythema in the affected region may also be present in the cases with OO [5,9]; however, these classic features of the lesion may not be present in all patients with OO. It has been reported that, among patients with OO, the frequency of presentation with nocturnal pain is 69%, and the rate for a favorable response to the NSAIDs is 72% [3]. In patients who do not provide this classical anamnesis and do not respond adequately to prostaglandin inhibition, as in the present patient, the diagnosis of OO should not be excluded.

Radiography, CT, MRI and bone scintigraphy are the imaging modalities used for the diagnosis of OO [3,6]. The tumor may be of the cancellous, cortical, or subperiosteal subtype with respect to its location within the bone [10]. Most foot OOs are reported to be cancellous or subperiosteal where the periosteal reaction is minimal or absent, making the radiographic diagnosis difficult [6]. CT excellently shows the typical bony features of the lesion and is regarded as the method of choice in the diagnosis of OO. MRI, which is the most used imaging modality for the investigation of foot and ankle pain, has a lower sensitivity and specificity in the diagnosis of OO compared to that of CT. Although it is superior to CT in the diagnosis of impingement, osteochondral defects, and ligamentous pathologies, MRI has been shown to be insufficient in the diagnosis of OO. The sensitivity of bone scintigraphy in the diagnosis of OO is comparable to that of CT, but the lack of its specificity significantly limits its use for this purpose [3,6]. In our case, radiographs revealed a seemingly silent sclerotic lesion without an accompanying periosteal reaction. As we had focused on the degenerative disease involving the interphalangeal joint in the initial evaluation, and expected to find an inflammatory soft tissue pathology in the second evaluation, we could not reach the correct diagnosis until a foot CT revealed the pathognomonic lucent nidus surrounded by a sclerotic rim.

Although surgical excision is the traditionally adopted treatment of OOs, imaging-guided laser and radiofrequency ablation have gained significant popularity in recent years [11,12]. CT-guided laser and radiofrequency ablation have been shown to be safe and effective treatment methods for foot OOs, with attention to lesions smaller than 1 cm in neurovascular structures or superficial areas due to the risk of soft tissue damage caused by thermal necrosis [11].

## 4 Conclusion

Although rare, OO may occur in the hallux. Even if the patient age, pain pattern, and radiographic findings do not exactly meet the classical definitions for OO, this tumor should always be included in the differential diagnosis list in patients presenting with foot pain.

## Sources of funding

This research did not receive any specific grant from funding agencies in the public, commercial, or not-for-profit sectors.

## Ethical approval

This article does not include research involving patients, so it does not require ethical approval.

## Consent

Informed consent for publication was obtained from the patient.

## Author contribution

MÖ: Acquisition of data, analysis and interpretation of data, drafting the article, final approval of the version to be published.

RPK: Acquisition of data.

BDŞ: Final approval of the version to be published.

ED: Analysis and interpretation of data.

All authors read and approved the final manuscript.

## Registration of research studies

N/A.

## Guarantor

Meltem Özdemir, MD.

## Provenance and peer review

Not commissioned, externally peer-reviewed.

## Declaration of Competing Interest

The authors declare that they have no conflict of interest.
